# Reward Optimization in the Primate Brain: A Probabilistic Model of Decision Making under Uncertainty

**DOI:** 10.1371/journal.pone.0053344

**Published:** 2013-01-22

**Authors:** Yanping Huang, Rajesh P. N. Rao

**Affiliations:** Department of Computer Science and Engineering, University of Washington, Seattle, Washington, United States of America; Radboud University Nijmegen, the Netherlands

## Abstract

A key problem in neuroscience is understanding how the brain makes decisions under uncertainty. Important insights have been gained using tasks such as the random dots motion discrimination task in which the subject makes decisions based on noisy stimuli. A descriptive model known as the drift diffusion model has previously been used to explain psychometric and reaction time data from such tasks but to fully explain the data, one is forced to make ad-hoc assumptions such as a time-dependent collapsing decision boundary. We show that such assumptions are unnecessary when decision making is viewed within the framework of partially observable Markov decision processes (POMDPs). We propose an alternative model for decision making based on POMDPs. We show that the motion discrimination task reduces to the problems of (1) computing beliefs (posterior distributions) over the unknown direction and motion strength from noisy observations in a Bayesian manner, and (2) selecting actions based on these beliefs to maximize the expected sum of future rewards. The resulting optimal policy (belief-to-action mapping) is shown to be equivalent to a collapsing decision threshold that governs the switch from evidence accumulation to a discrimination decision. We show that the model accounts for both accuracy and reaction time as a function of stimulus strength as well as different speed-accuracy conditions in the random dots task.

## Introduction

Animals are constantly confronted with the problem of making decisions given noisy sensory measurements and incomplete knowledge of their environment. Making decisions under such circumstances is difficult because it requires (1) inferring hidden states in the environment that are generating the noisy sensory observations, and (2) determining if one decision (or action) is better than another based on uncertain and delayed reinforcement. Experimental and theoretical studies [1]–[6] have suggested that the brain may implement an approximate form of Bayesian inference for solving the hidden state problem. However, these studies typically do not address the question of how probabilistic representations of hidden state are employed in action selection based on reinforcement. Daw, Dayan and their colleagues [7], [8] explored the suitability of decision theoretic and reinforcement learning models in understanding several well-known neurobiological experiments. Bogacz and colleagues proposed a model that combines a traditional decision making model with reinforcement learning [9] (see also [10]). Rao [11] proposed a neural model for decision making based on the framework of partially observable Markov decision processes (POMDPs) [12]; the model focused on network implementation and learning but assumed a deadline to explain the collapsing decision threshold. Drugowitsch et al. [13] sought to explain the collapsing decision threshold by combining a traditional drift diffusion model with reward rate maximization. Other recent studies have used the general framework of POMDPs to explain experimental data in decision making tasks such as those involving a stop-signal [14], [15] and different types of prior knowledge [16].

In this paper, we derive from first principles a POMDP model for the well-known random dots motion discrimination task [17]. We show that the task reduces to the problems of (1) computing beta-distributed beliefs over the unknown direction and motion strength from noisy observations, and (2) selecting actions based on these beliefs in order to maximize the expected sum of future rewards. Without making ad-hoc assumptions such as a hypothetical deadline, a collapsing decision threshold emerges naturally via expected reward maximization. We present results comparing the model's predictions to experimental data and show that the model can explain both reaction time and accuracy as a function of stimulus strength as well as different speed-accuracy conditions.

## Methods

### POMDP framework

**Figure 1:**
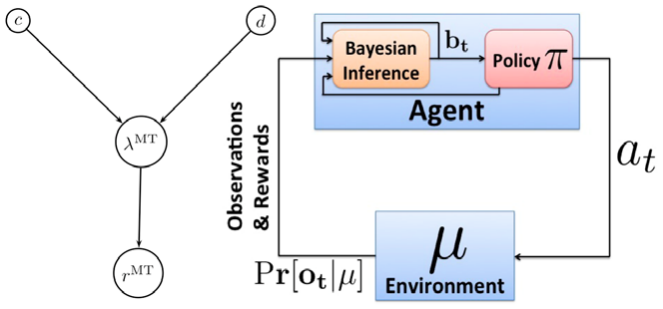
POMP Framework for Decision Making. *Left:* The graphical model representing the probabilistic relationship between random variables 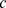, 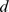, 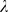 and 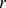. In the POMDP model, the hidden state 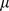 corresponds to coherence 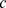 and direction 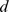 jointly. The observation 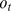 corresponds to MT response 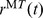. The relations between these variables are summarized in table 1. *Right:* In order to solve a POMDP problem, the animal maintains a belief 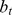, which is a posterior probability distribution over hidden states 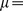 of the world given observations 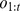. At a current belief state 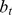, an action is selected according to the learned policy 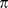, which maps belief states to actions.

We model the random dots motion discrimination task as a POMDP. The POMDP framework assumes that at any particular time step, the environment is in a particular *hidden* state, 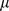, that is not directly accessible to the animal. This hidden state however can be inferred by making a sequence of sensory measurements. At each time step 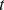, the animal receives a sensory measurement (observation), 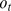, from the environment, which is determined by an 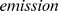 probability distribution 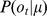. Since the hidden state 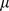 is unknown, the animal must maintain a *belief* (posterior probability distribution) over the set of possible states given the sensory observations seen so far: 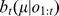, where 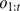 represents the sequence of observations that the animal has accumulated so far. At each time step, an action (decision) 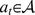 made by the animal can affect the environment by changing the current state to another according to a 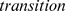 probability distribution 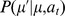 where 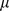 is the current state, and 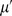 is a new state. The animal then gets a reward 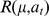 from the environment, depending on the current state and the action taken. During training, the animal learns a policy, 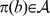, which indicates which action 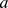 to perform for each belief state 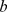. We make two main assumptions in the POMDP model. First, the animal uses Bayes rule to update its belief about the hidden state after each new observation 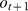: 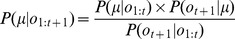. Second, the animal is trained to follow an *optimal policy* 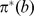 that maximizes the animal's expected total future reward in the task. Figure 1 illustrates the decision making process using the POMDP framework. Note that in the decision making tasks that we model in this paper, the hidden state 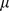 is fixed by experimenters within a trial and thus there is no transition distribution to include in the belief update equation. In general, the hidden state in a POMDP model follows a Markov chain, making the observations 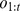 temporally correlated.

### Random dots task as a POMDP

We now describe how the general framework of POMDPs can be applied to the random dots motion discrimination task as shown in Figure 1. In each trial, experimenter chooses a fixed direction 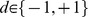 corresponding to leftward and rightward motion respectively, and a stimulus strength (motion coherence) 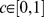, where 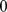 corresponds to completely random motion and 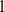 corresponds to 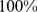 coherent motion (i.e., all dots moving in the same direction). Intermediate values of 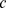 represent a corresponding fraction of dots moving in the coherent direction (e.g., 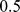 represents 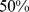 coherent motion). The animal is shown a movie of randomly moving dots, a fraction 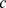 of which are moving in the same direction 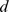.

In a given trial, neither the direction 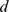 nor the coherence 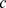 is known to the animal. We therefore regard 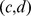 as the joint hidden environment state 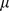 in the POMDP model. Neurophysiological evidence suggests that information regarding random dot motion is received from neurons in cortical area MT [18]–[21]. Therefore, following previous models (e.g., [22]–[24]), we define the observation model 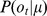 in the POMDP as a function of the responses of MT neurons. Let the firing rate of MT neurons preferring rightward and leftward direction be 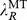 and 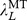 respectively. We can define:
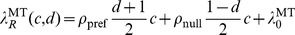


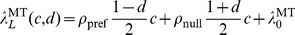
(1)where 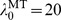 spikes/second is the average spike rate for 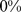 coherent motion stimulus, and 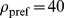 and 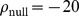 are the “drive” in the preferred and null directions respectively. These constants (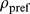, 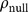 and 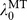) are based on fits to experimental data as reported in [23], [25]. Let 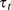 be the elapsed time between time steps 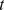 and 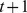. Then, the number of spikes emitted by MT neurons 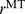 within 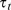 follows a Poisson distribution:
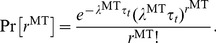
(2)We define the observation 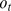 at time 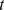 as the spike count from MT neurons preferring rightward motion, given the total spike count from rightward and leftward-preferring neurons, i.e., the observation is a conditional random variable 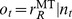 where 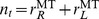. Then 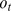 follows a stationary Binomial distribution 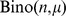. Note that the duration of each POMDP time step need not be fixed, and we can therefore adjust 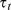 such that 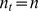 for some fixed 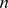, i.e., the animal updates the posterior distribution over hidden state each time it receives 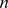 spikes from the MT population. 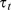 is exponentially distributed, and the standard deviation of 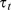 will approach zero as 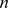 increases. When 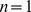, 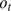 becomes an indicator random variable representing whether a spike was emitted by a rightward motion preferring neuron or not.

It can be shown [26] that 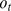 follows a Binomial distribution 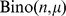 with
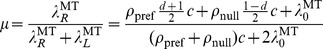
(3)

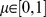
 represents the probability that the MT neurons favoring rightward movement will spike given that there is a spike in the MT population. Since 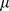 is a joint function of 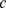 and 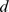, we could equivalently regard it as the hidden state of our POMDP model: 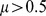 indicates rightward direction (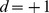) while 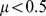 indicates the opposite direction (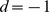). The coherence 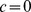 corresponds to 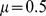 while 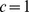 corresponds to the two extreme values 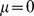 or 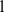 for direction 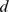 being left or right respectively. Note that both direction 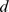 and coherence 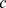 are unknown to the animal in the experiments, but they are held constant within a trial.

### Bayesian inference of hidden state

Given the framework above, the task of deciding the direction of motion of the coherently moving dots is equivalent to the task of deciding whether 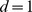 or not, and deciding when to make such a decision. The POMDP model makes decisions based on the “belief” state 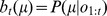, which is the posterior probability distribution over 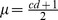 given a sequence of observations 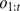:
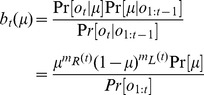
(4)where 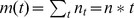, 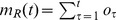, and 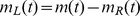. To facilitate the analysis, we represent the prior probability 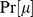 as a beta distribution with parameters 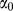 and 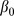. Note that the beta distribution is quite flexible: for example, a uniform prior can be obtained using 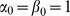. Without loss of generality, we will fix 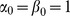 throughout this paper. The posterior distribution can now be written as:
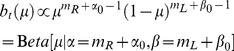
(5)The belief state 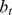 at time step 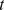 thus follows a beta distribution with two parameters 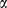 and 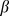 as defined above. Consequently, the posterior probability distribution over 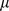 depends only on the number of spikes 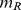 and 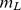 for rightward and leftward motion respectively. These in turn determine 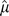 and 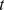, where
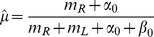
(6)is the point estimator of 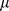, and 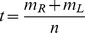. The animal only needs to keep track of 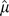 and 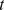 in order to encode the belief state 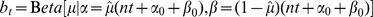. After marginalizing over coherence 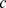, we have the posterior probability over direction 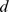:
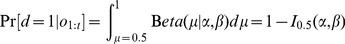
(7)

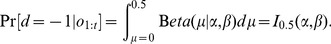
(8)where 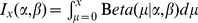 is the regularized incomplete beta function.

### Actions, rewards, and value function

The animal updates its belief after receiving the current observation 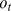, and chooses one of the three actions (decisions) 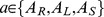, denoting rightward eye movement, leftward eye movement, and sampling (i.e., waiting for one more observation) respectively. The model assumes the animal receives rewards 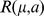 as follows (rewards are modeled using real numbers). When the animal makes a correct choice, 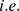, a rightward eye movement 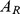 when 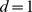 (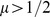) or a leftward eye movement 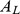 when 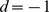 (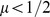), the animal receives a positive reward 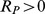. The animal receives a negative reward (i.e., penalty) or nothing when an incorrect action is chosen 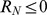. We further assume that the animal is motivated by hunger or thirst to make a decision as quickly as possible. This is modeled using a unit penalty 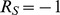 for each observation the animal makes, representing the cost the animal needs to pay when choosing the sampling action 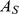.

Recall that a belief state 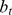 is determined by the parameters 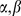. The goal of the animal is to find an optimal “policy” 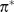 that maximizes the “value” function 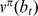, defined as the expected sum of future rewards given the current belief state:
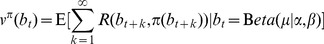
(9)where the expectation is taken with respect to all future belief states 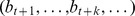. The reward term 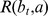 above is the expected reward for the given belief state and action:
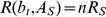
(10)

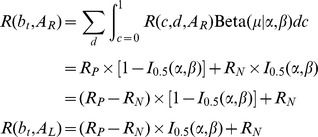
The above equations can be interpreted as follows. When 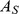 is selected, the animal receives 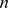 more samples at a cost of 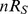. When 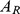 is selected, the expected reward 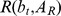 depends on the probability density function of the hidden parameter 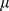 given belief state 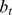. With probability 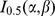, the true parameter 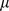 is less than 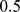, making 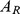 an incorrect decision with penalty 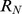, and with probability 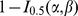, action 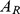 is correct, earning the reward 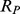.

### Finding the optimal policy

A policy 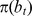 defines a mapping from a belief state to one of the available actions 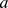. A method for learning a POMDP policy by trial and error using the method of temporal difference (TD) learning was suggested in [11]. Here, we derive a policy from first principles and compare the result with behavioral data.

One standard way [12] to solve a POMDP is to first convert it into a Markov Decision Process (MDP) over belief state, and then apply standard dynamical programming techniques such as value iteration [27] to compute the value function in equation 9. For the corresponding *belief MDP*, we need to define the transition probabilities 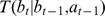. When 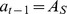, the belief state can be updated using the previous belief state and current observation based on Bayes' rule:
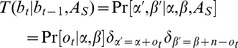
(11)for all 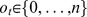. In the above equation, 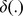 is the Kronecker delta, and 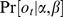 is the expected value of the likelihood function 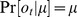 over the posterior distribution 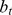:
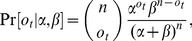
(12)which is a stationary distribution independent of time 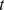. When the selected action is 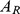 or 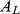, the animal stops sampling and makes an eye movement. To account for such cases, we include an additional state 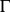, representing a terminal state, with zero reward 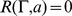 and absorbing behavior, 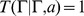 for all actions 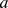. Formally, the transition probabilities with respect to the absorbing (termination) state are defined as 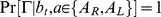 for all 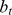, indicating the end of a trial.

Given the time-independent belief state transition 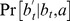, the optimal value 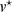 and policy 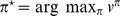 can be obtained by solving Bellman's equation:
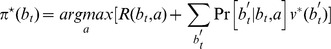


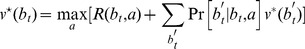
(13)


Before we proceed to results from the model, we note that the one-step belief transition probability matrix 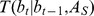 with 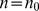 can be shown be mathematically equivalent to the 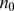-steps transition matrix 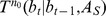 with 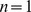. The solution to Bellman's equation 13 is independent of 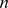. Therefore, unless otherwise mentioned, the results are based on the most general scenario where the animal needs to select an action whenever a new spike is received, 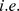, 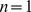.

We summarize the model variables as well as their statistical relationships in table 1.

**Table 1:** Summary of model variables and paramters.

POMDP Variables	Descriptions
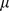	The hidden variable of POMDP, 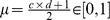 . In the random dots task, 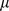 is a constant over time
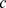	The coherence (motion strength) of the random dots task. 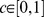 . 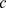 is fixed during a task.
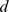	The underlying direction of the random dots task. 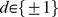 . 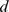 is fixed during a task.
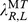	The average spike rate of MT neurons preferring rightward or leftward direction, respectively, as a function of both coherence 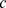 and 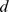 described in equations 1.
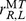	The number of spikes emitted by MT neurons preferring rightward or leftward direction, respectively during one POMDP step. 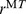 follows a Poisson distribution with mean 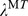
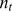	Total number of spikes emitted by MT neurons during one POMDP step. 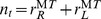
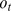	The noisy observation at time step t, which is a conditional random variable 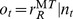 following a Binomial distribution 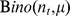 . Note that 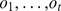 are conditional dependent of each other given the hidden variable 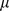
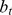	The belief (posterior distribution) 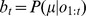 . With a beta-distributed initial belief 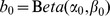 , 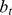 is also beta distributed due to the binomial distributed emission probability 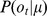 . Without loss of generality, 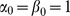 throughout the paper.
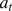	Action chosen by the animal at time 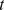 . 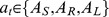 .
Model Parameters	
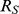	A negative reward associated with the cost of an observation.
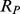	A positive reward associated with a correct eye movement.
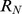	A negative reward associated with an incorrect eye movement.
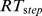	The duration of a single observation, the real elapsed time per POMDP step. Only used to translate the number of POMDP time steps to real elapsed time when comparing with experimental data.
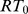	Non-decision residual time. Both 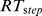 and 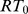 are obtained from a linear regression to compare model predictions (in unit of POMDP steps) with animals' response time (in unit of seconds), independent of the POMDP model.

## Results

### Optimal value function and policy

**Figure 2:**
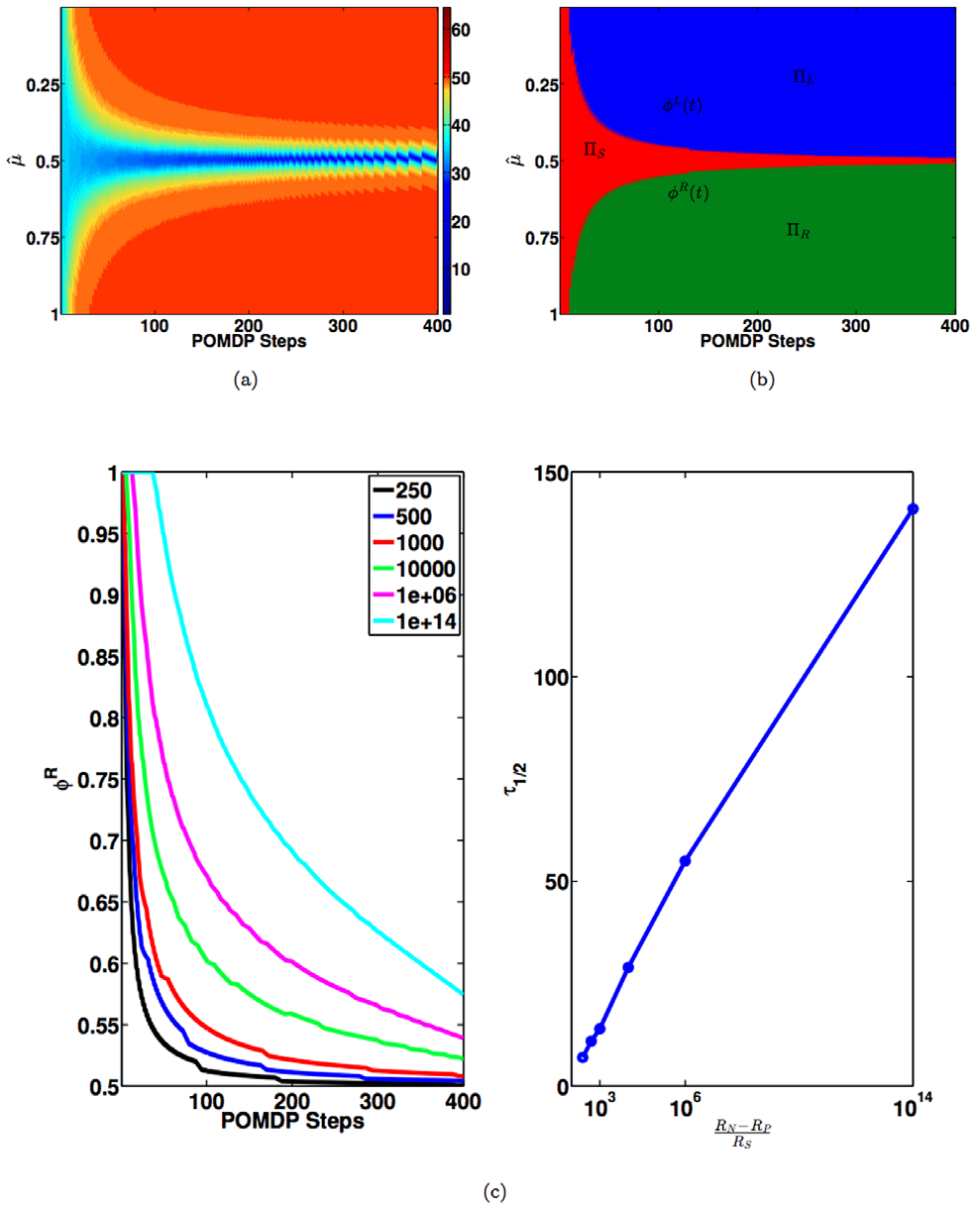
Optimal Value and Policy for the Random Dots Task. (a) Optimal value as a joint function of 
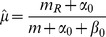
 and the number of POMDP steps 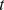. (b) Optimal Policy as a function of 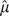 and the number of POMDP steps 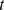. The boundaries 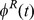 and 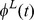 divide the belief space into three areas: 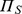 (red), 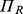 (green), and 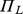 (blue), each of which represents belief states whose optimal actions are 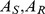 and 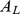 respectively. Model parameters: 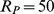, 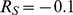, and 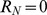. (c) *Left:* The rightward decision boundary 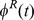 for different values of 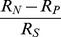. *Right:* The half time 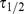 of 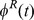 for different values of 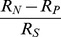, where 
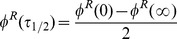
.

Figure 2 (a) shows the optimal value function computed by applying value iteration [27] to the POMDP defined in the Methods and Analysis section, with parameters 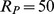, 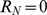, and 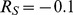. The 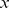-axis of Figure 2 (a) represents the total number of observations 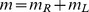 encountered thus far, which is equal to the elapsed time 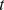 in the trial. The 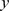-axis represents the ratio 
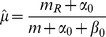
, which is the estimator of the hidden parameter 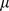. In general, the model predicts a high value when 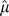 is close to 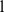 or 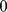, or equivalently, when the estimated coherence is close to 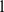. This is because at these two extremes, selecting the appropriate action has a high probability of receiving a large positive reward 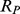. On the other hand, for 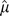 near 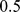 (estimated 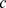 near 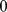), choosing 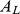 or 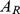 in these states has a high chance of resulting in an incorrect decision and a large negative reward 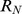 (see [11] for a similar result using a different model and under the assumption of a deadline). Thus, belief states with 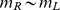 have a much lower value compared to belief states with 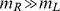 or 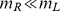.

Figure 2 (b) shows the corresponding optimal policy 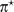 as a joint function of 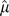 and 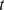. The optimal policy 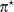 partitions the belief space into three regions: 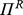, 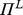, and 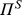, representing the set of belief states preferring actions 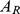, 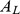 and 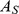 respectively. Let 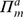 be the set of belief states preferring action 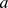 after 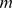 observations, for 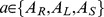 and 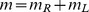. Early in a trial, when 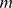 is small, the model selects the sampling action 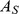 regardless of the value of 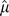. This is because for small 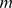, the variance of the point estimator 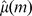 is high. For example, even when 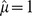 when 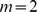, the probability that the true 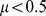 is still high. The sampling action 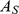 is required to reduce this variance by accruing more evidence. As 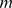 becomes larger, the variance of 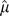 decreases, and the deviation between 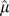 and the true value of 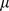 diminishes by the law of large numbers. Consequently, the animal will pick action 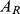 even when 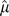 is only slightly above 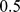. This gradual decrease in the threshold over time for choosing the overt actions 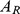 or 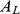 has been called a “collapsing bound” in the decision making literature [28]–[30].

The optimal policy 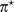 is entirely determined by three reward parameters 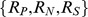. At a given belief state, 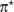 picks one of the three available actions that leads to the largest expected future reward. Thus, the choice is determined by the relative, not the absolute, value of the expected future reward for the different actions. From equation 10, we have
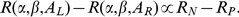
(14)If we regard the sampling penalty 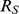 as specifying the unit of reward, the optimal policy 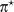 is determined by the ratio 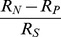 alone. Figure 2 (c) shows the relationship between 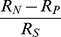 and the optimal policy 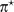 by showing the rightward decision boundaries 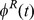 for different values of 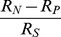. As 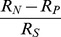 increases (e.g., by making the sampling cost 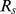 smaller), the boundary 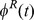 gradually moves towards the upper right corner, giving the animal more time to make decisions which results in more accurate decisions. To better understand this relationship, we fit the decision boundary to a hyperbolic function:
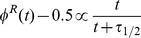
(15)We find that 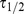 exhibits nearly logarithmic growth with 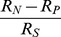. Interestingly, a collapsing bound is obtained even with extremely small 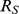 because the goal is reward maximization across trials: it is better to terminate a trial and accrue reward in future trials than to continue sampling noisy (possibly 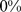 coherent) stimuli.

### Model predictions: psychometric function and reaction time

**Figure 3:**
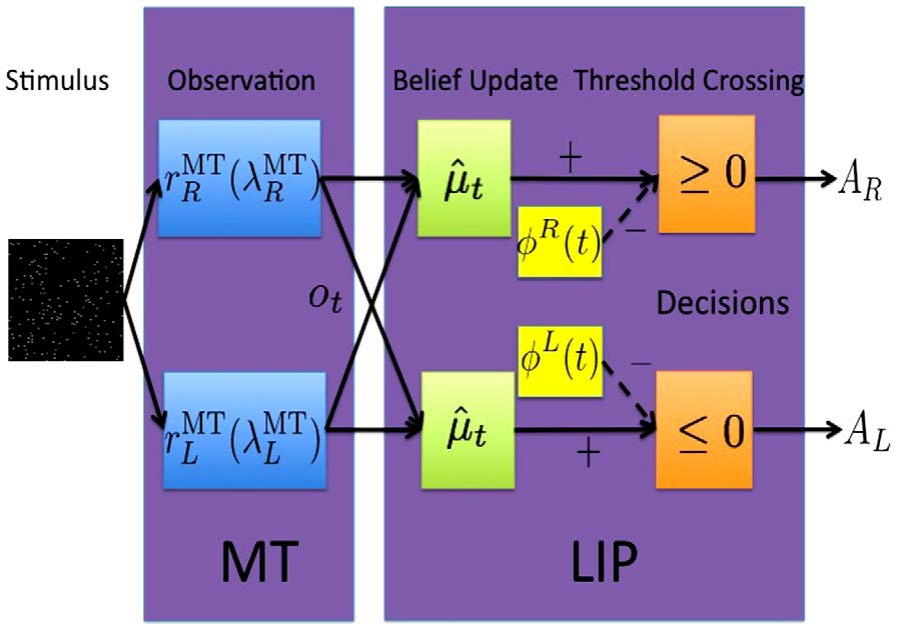
Relationship between Model and Neural Activity. The input to the model is a random dots motion sequence. Neurons in MT with tuning curves 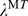 emit 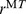 spikes at time step 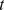, which constitutes the observation 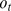 in the POMDP model. The animal maintains the belief state 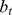 by computing 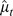 (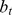 can be parameterized by 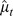 and 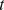 - see text). The optimal policy is implemented by selecting rightward eye movement 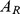 when 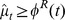, or equivalently, when 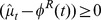 (and likewise for leftward eye movement 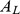).

We compare predictions of the model based on the learned policy 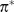 with experimental data from the reaction time version (rather than the fixed duration version) of the motion discrimination task [31]. As illustrated in Figure 3, the model assumes that motion information regarding the random dots on the screen is processed by MT neurons. These neurons provide the observations 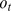 (and 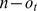) to right- and left-direction coding LIP neurons, which maintain the belief state 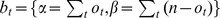. Actions are selected based on the optimal policy 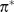. If 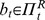 or 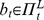, the animal makes a rightward or leftward decision respectively and terminates the trial. When 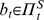, the animal chooses the sampling action and gets a new observation 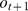.

The performance on the task using the optimal policy 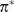 can be measured in terms of both the accuracy of direction discrimination (the so-called psychometric function), and the reaction time required to reach a decision (the chronometric function). In this section, we derive the expected accuracy and reaction time as a function of stimulus coherence 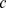, and compare them to the psychometric and chronometric functions of a monkey performing the same task [31].

The sequence of random variables 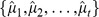 forms a (non-stationary) Markov chain with transition probabilities determined by equation 11. Let 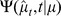 be the joint probability that the animal keeps selecting 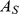 until time step 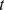:
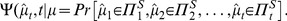
(16)At 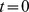, the animal will select 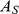 regardless of 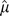 under 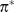, making 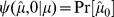. At 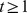, 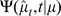 can be expressed recursively as:
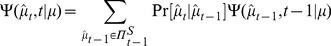
(17)


Let 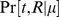 and 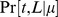 be the joint probability mass functions that the animal makes a right or left choice at time 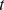, respectively. These correspond to the probability that the point estimator 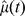 crosses the boundary of 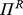 or 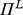 for the first time at time 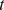:
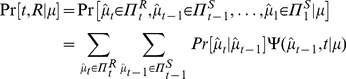
(18)

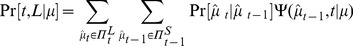
(19)


The probabilities of making rightward or leftward eye movement are the marginal probabilities summing over all possible crossing times: 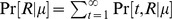 and 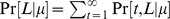. When the underlying motion direction is rightward, 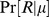 represents the accuracy of motion discrimination and 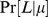 represents the error rate. The mean reaction times for correct and error choices are the expected crossing times over the conditional probability that the animal makes decision 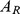 and 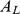 respectively at time 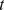:
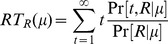
(20)

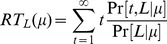
(21)


**Figure 4:**
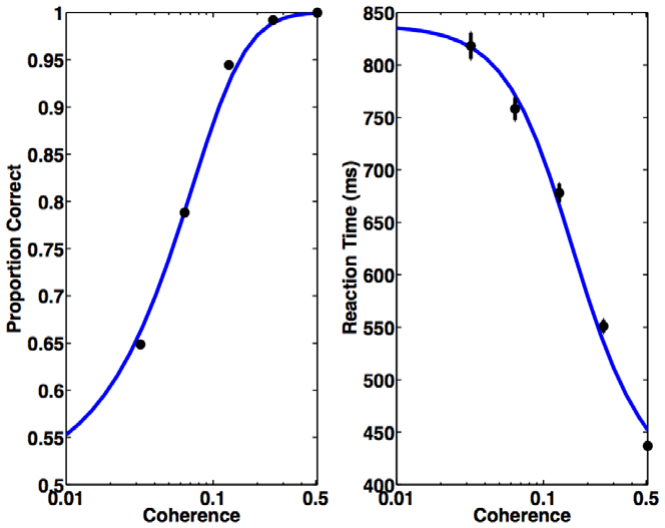
Comparison of Performance of the Model and Monkey. Black dots with error bars represent a monkey's decision accuracy and reaction time for correct trials. Blue solid curves are model predictions (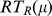 and 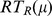 in the text) for parameter values 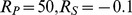, and 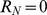. Monkey data from [31].

The left panel of Figure 4 shows performance accuracy as a function of motion strength 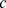 for the model (solid curve) and a monkey (black dots). The model parameters are the same as those in Figure 2, obtained using a binary search within 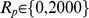 with a minimum step size 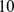.

The right panel of Figure 4 shows for the same model parameters the predicted mean reacton time 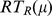 for correct choices as a function of coherence 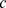 (and fixed direction 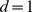) for the model (solid curve) and the monkey (black dots). Note that 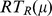 represents the expected number of POMDP time steps for making a rightward eye movement 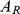. It follows from the Poisson spiking process that the duration of each POMDP time step follows a exponential distribution with its expectation proportional to 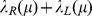. In order to make a direct comparison to the monkey data 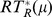, which is in units of real time, a linear regression was used to to determine the duration 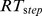 of a single observation and the onset of decision time 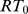:
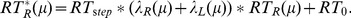
(22)Note that the reaction time in a trial is the sum of decision time plus the non-decision delays whose properties are not well understood. The offset 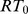 represents the non-decision residual time. We applied the experimental mean reaction time reported in [31] with motion coherence 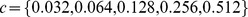 to compute the two coefficients 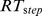 and 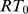. The unit duration per POMDP step 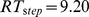 ms/step, and the offset 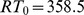 ms, which is comparable to the 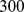 ms non-decision time on average reported in the literature [23], [32].

**Figure 5:**
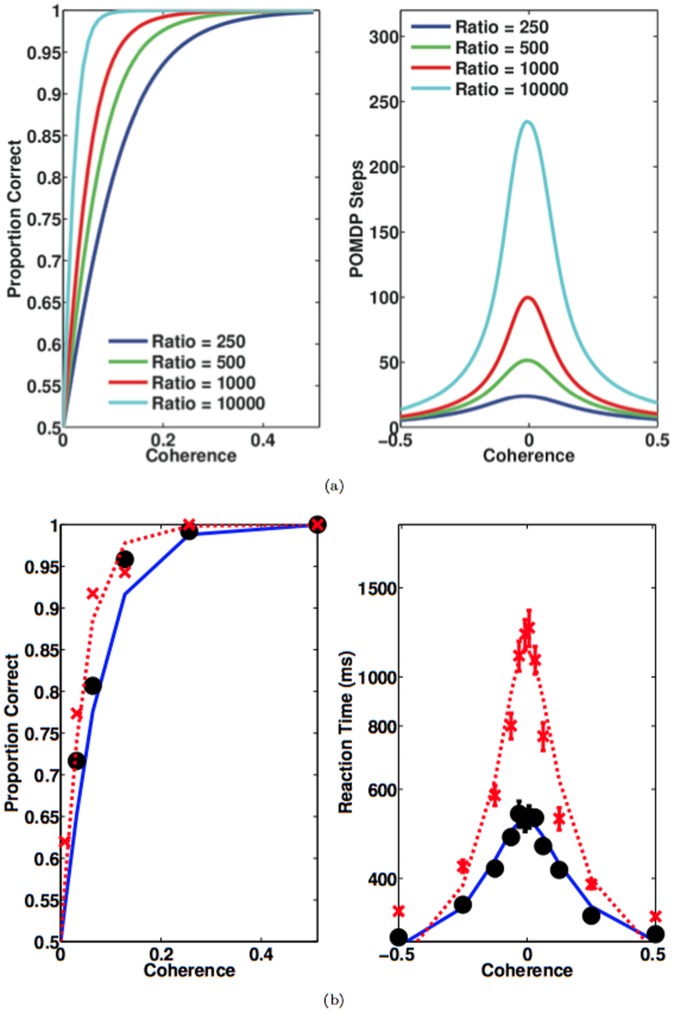
Effect of 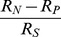 on speed-accuracy tradeoff. (a) Model predictions of psychometric and chronometric functions for different values of 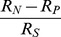. (b) Comparison of model predictions and experimental data for different speed-accuracy regimes. The black dots represent the response time and accuracy of a human subject in the direction discrimination task under normal speed conditions, while the red crosses represent data with a slower speed instruction. The model predictions are plotted as black solid curves (with 
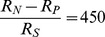
) and red dashed lines (
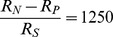
), respectively. The per-step duration and non-decision residual time are fixed to be the same for both conditions: 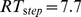 ms/step, and 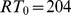 ms. Human data are from human subject LH in [33].

There is essentially one parameter in our model needed to fit the experimental accuracy data, namely, the reward ratio 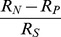. The other two parameters 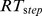 and 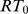 are independent of the POMDP model, and are used only to translate the POMDP time steps into real elapsed time. This reward ratio has direct physical interpretation and can be easily manipulated by the experimenters. For example, changing the amount of awards for the correct/incorrect choices, or giving subjects different speed instructions will effectively change 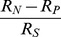. In Figure 5 (a), we show performance accuracies 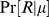 and predicted mean reaction time 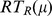 with different values of 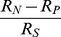. With fixed 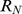 and 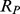, decreasing 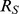 makes the observations more affordable and allows subjects to accumulate more evidence, in turn leads to a longer decision time and higher accuracy. Our model thus provides a quantitative framework for predicting the effects of reward parameters on the accuracy and speed of decision making. To test our theory, we compare the model predictions with the experimental data from a human subject, reported by Hanks et al [33], under different speed-accuracy regimes. In their experiments, human subjects were instructed to perform the random dots task under different speed-accuracy conditions. The red crosses in Figure 5 (b) represent the response time and accuracy of a human subject in the direction discrimination task with instructions to perform the task more carefully at a slower speed, while the black dots represent the task under normal speed conditions. The slower speed instruction encourages human subjects to accumulate more observations before making the final decision. In the model, this amounts to reducing the negative cost associated with each sample 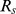. Indeed, this tradeoff between speed and accuracy was consistent with predicted effects of changing the reward ratio. We first fit the model parameters to experimental data under normal speed conditions, based on fitting 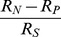, 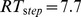 ms/step, and 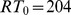 ms (Figure 5 (b), black solid curves). The red dashed lines shown in Figure 5 (b) are model fits to the data under slower speed instruction. There is just one degree of freedom in this fit, as all model parameters except the reward ratio were fixed to the values used to fit data in the normal speed regime.

### Neural response during direction discrimination task

From Figure 2 (b), it is clear that for the random dots task, the animal does not need to store the whole two dimensional optimal policy but only the two one-dimensional decision boundaries 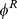 and 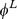. This naturally suggests a neural mechanism for decision making similar to that in drift diffusion models: LIP neurons compute the belief state from MT responses and employ divisive normalization to maintain the point estimate 
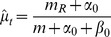
. We now explore the hypothesis that the response of LIP neurons represents the difference between 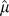 and the optimal decision threshold 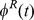. In this model, a rightward eye movement is initiated only when the difference 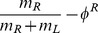 reaches a fixed bound (in this case, 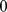). Therefore, we modeled the firing rates in the lateral intraparietal area (LIP) 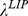 as:
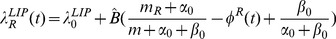
(23)where 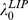 is the spontaneous firing rate for LIP neurons. Since 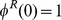, a constant 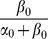 is added to make 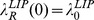. 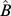 represents the termination bound; 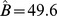 spikes s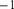 from [30]. The firing rate 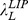 is defined similarly.

**Figure 6:**
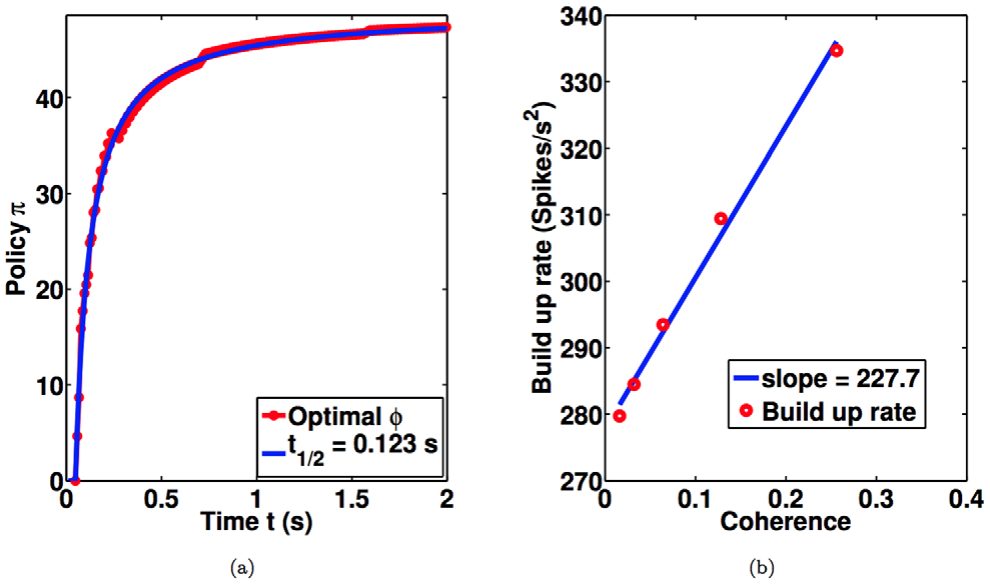
Comparison of Model and Neural Responses. (a) Model response to 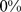 coherence motion is shown in red. Blue curve depicts a fit using a hyperbolic function 
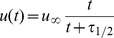
 where 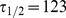 ms, which is comparable to the value of 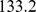 ms estimated from neural data [30]. (b) The first 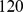 ms of decision time was used to compute the buildup rate from the model response following the procedure in [30]. The red points show model buildup rates estimated for each coherence value. The effect of a unit change in the coherence on buildup rate can be estimated from the slope of the blue fitted line: this value, 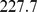 spike s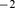 coh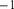, is similar to the corresponding value 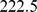 spike s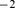 coh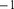 estimated from neural data [30].

The above model makes two testable predictions about neural responses in LIP. The first is that the neural response to 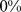 coherent motion (the so called “urgency” signal [30], [34]) encodes the decision boundary 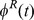 (or 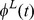 for leftward-preferring LIP neurons). In Figure 6a, we plot the model response to 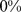 coherent motion, along with a fit to a hyperbolic function 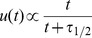, the same function that Churchland et al [30] used to parametrize the experimentally observed “urgency signal.” The parameter 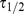 is the time taken to reach 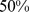 of the maximum. The estimate of 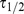 for the model from Figure 6 (a) is 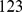 ms, which is consistent with the 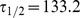 ms estimated from neural data [30].

The second prediction concerns the buildup rate (in units of spikes s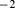 coh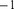) of the LIP firing rates. The buildup rate of LIP at each motion strength is calculated from the slope of a line fit to model LIP firing rate during the first 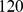 ms of decision time. As shown in Figure 6 (b), buildup rates scaled approximately linearly as a function of motion coherence. The effect of a unit change in coherence on the buildup rate can be estimated from the slope of the fitted line to be 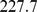 spike s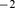 coh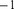, similar to what has been reported in the literature [30] (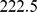 spike s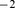 coh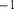).

## Discussion

The random dots motion discrimination task has provided a wealth of information regarding decision making in the primate brain. Much of this data has previously been modeled using the drift diffusion model [35], [36], but to fully account for the experimental data, one has to sometimes use ad-hoc assumptions. This paper introduces an alternative model for explaining the monkey's behavior based on the framework of partially observable Markov decision processes (POMDPs).

We believe that the POMDP model provides a more versatile framework for decision making compared to the drift diffusion model, which can be viewed as a special case of sequential statistical hypothesis testing (SSHT) [37]. Sequential statistical hypothesis testing assumes that the stimuli (observations) are independent and identically distributed whereas the POMDP model allows observations be temporally correlated. The observations in the POMDP are conditionally independent given the hidden state 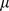, which evolves according to a Markov chain. Thus, the POMDP framework for decision making [11], [14], [16], [38], [39] can be regarded as a strictly more general model than the SSHT models. We intend to explore the applicability of our POMDP model to time-dependent stimuli, such as temporally dynamic attention [40] and temporally blurred stimulus representations [41] in future studies.

Another advantage of a POMDP model is that the model parameters have direct physical interpretations and can be easily manipulated by the experimenter. Our analysis shows that the optimal policy is fully determined by the reward parameters 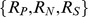. Thus, the model psychometric and chronometric functions, which are derived from the optimal policy, are also fully determined by these model parameters. Experimenters can control these reward parameters by changing the amount of awards for the correct/incorrect choices, or by giving subjects different speed instructions. This allows our model to make testable predictions, as demonstrated by the effects of the change in the reward ratios on the speed-accuracy trade-off. It should be noted that these reward parameters can be subjective and may vary from individual to individual. For example, 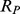 can be directly related to the external food or juice reward provided by the experimenter while 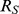 may be linked to internal factors such as degree of hunger or thirst, drive, and motivation. The precise relationship between these reward parameters and the external reward/risk controlled by the experimenter remains unknown. Our model thus provides a quantitative framework for studying this relationship between internal reward mechanisms and external physical reward.

The proposed model demonstrates how the monkey's choices in the random dots task can be interpreted as being optimal under the hypothesis of reward maximization. The reward maximization hypothesis has previously been used to explain behavioral data from conditioning experiments [8] and dopaminergic responses under the framework of temporal difference (TD) learning [42]. Our model extends these results to the more general problem of decision making under uncertainty. The model predicts psychometric and chronometric functions that are quantitatively close to those observed in monkeys and humans solving the random dots task.

We showed through analytical derivations and numerical simulation that the optimal threshold for selecting overt actions is a declining function of time. Such a collapsing decision bound has previously been obtained for decision making under a deadline [11], [29]. It has also been proposed as an ad-hoc mechanism in drift diffusion models [28], [30], [43] for explaining finite response time at zero percent coherence. Our results demonstrate that a collapsing bound emerges naturally as a consequence of reward maximization. Additionally, the POMDP model readily generalizes to the case of decision making with arbitrary numbers of states and actions, as well as time-varying state.

Instead of traditional dynamic programming techniques, the optimal policy 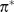 and value 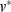 can be learned via Monte Carlo approximation-based methods such as temporal difference (TD) learning [27]. There is much evidence suggesting that the firing rate of midbrain dopaminergic neurons might represent the reward prediction error in TD learning. Thus, the learning of value and policy in the current model could potentially be implemented in a manner similar to previous TD learning models of the basal ganglia [8], [9], [11], [42].
